# Kinetics of Oxygen Exchange and N_2_O Decomposition Reaction over MeO_x_/CeO_2_ (Me = Fe, Co, Ni) Catalysts

**DOI:** 10.3390/ma16030929

**Published:** 2023-01-18

**Authors:** Ekaterina Sadovskaya, Larisa Pinaeva, Valerii Skazka, Igor Prosvirin

**Affiliations:** 1 Boreskov Institute of Catalysis SB RAS, 5, Lavrentiev Ave., 630090 Novosibirsk, Russia; 2 Department of Mathematics and Mechanics, Novosibirsk State University, 2, Pirogov Str., 630090 Novosibirsk, Russia

**Keywords:** temperature programmed isotope exchange, oxygen mobility, surface, Me–Ce interface

## Abstract

MeO_x_/CeO_2_ (Me = Fe, Co, Ni) samples were tested in an ^18^O_2_ temperature-programmed isotope exchange and N_2_O decomposition (deN_2_O). A decrease in the rate of deN_2_O in the presence of oxygen evidences the competitive adsorption of N_2_O and O_2_ on the same sites. A study of isotope oxygen exchange revealed dissociative oxygen adsorption with the subsequent formation of surface oxygen species. The same species, more probably, result from N_2_O adsorption and the following N_2_ evolution to the gas phase. We supposed the same mechanism of O_2_ formation from surface oxygen species in both reactions, including the stages responsible for its mobility. A detailed analysis of the kinetics of isotope exchange has been performed, and the rates of one-atom (*R*^I^) and two-atom (*R*^II^) types of exchange were evaluated. The rate of the stage characterizing the mobility of surface oxygen was calculated, supposing the same two-step mechanism was relevant for both types of exchange. The effect of oxygen mobility on the kinetics of deN_2_O was estimated. An analysis of the possible pathways of isotope transfer from MeO_x_ to CeO_x_ showed that direct oxygen exchange on the Me–Ce interface makes a valuable contribution to the rate of this reaction. The principal role of the Me–Ce interface in deN_2_O was confirmed with independent experiments on FeO_x_/CeO_2_ samples with a different iron content.

## 1. Introduction

N_2_O is one of the side products of NH_3_ oxidation to nitric acid over Pt–Rh gauzes; its emissions into the atmosphere are strictly regulated. It is commonly considered that N_2_O decomposition (deN_2_O) is initiated by the interaction of gas-phase N_2_O with an active site S (oxygen vacancies, in our case), resulting in the gas-phase N_2_ and adsorbed oxygen species (O-S):

(1) N_2_O + S ↔ N_2_O-S
(2) N_2_O-S → N_2_ + O-S

The formation of O_2_ can proceed by either direct recombination of two O-S species [1,2]:

(3a) 2O-S ↔ O_2_ + 2S

or by direct reaction of gas-phase N_2_O with O-S [3] described by the simplified reaction:

(3b) O-S + N_2_O → N_2_ + O_2_ + S

Reaction inhibition in oxygen presence was observed for most bulk oxide systems [1,2], which can be due to the competitive adsorption of N_2_O and oxygen on the same active sites. At 300–600 °C, oxygen desorption was shown to be the rate-controlling step of the reaction [4,5,6]. N_2_O decomposition in the ammonia burner with the catalyst located directly under Pt-Rh gauzes is also industrially applied as an attractive technology for N_2_O abatement. Such a catalyst operates at above 800 °C. Under these conditions, the importance of oxygen mobility for the deN_2_O activity was highlighted for La–Sr–Mn perovskite-type oxides [7] and supported MeO_x_/Al_2_O_3_(CeO_2_) (Me = Fe, Co, Ni) [8] and FeO_x_/(CeO_2_ + Al_2_O_3_) samples [9], while reaction retardation with oxygen was observed as well. The mechanism of oxygen formation through the recombination of two O-S species (step 3a) is unlikely to fit this case because oxygen species are strongly bonded with anion vacancies and cannot diffuse on the catalyst surface. Kondratenko et al. [10,11,12,13] showed that N_2_O decomposition over different Fe-containing oxidic systems used in a wide range of temperatures is better fitted using an intermediate scheme. With this scheme, O_2_ and N_2_O adsorb on the same active site S, as in the case of the traditional Langmuir-Hinshelwood mechanism. However, oxygen adsorption proceeds without dissociation, and the molecular form of adsorbed O_2_ (O_2_-S) has been formed by a N_2_O reaction with O-S:

(3c) O_2_-S ↔ O_2_ + S
(4) N_2_O + O-S → N_2_ + O_2_-S

However, samples with preferentially isolated active Fe sites (Fe-ZSM-5, Fe-silicalite, and BaFeAl_11_O_19_) have been studied, and non-dissociative O_2_ adsorption can be supposed in this case. Data from Boreskov and Muzykantov [14] show that at >~400 °C, oxygen dissociative adsorption proceeds on the surface of bulk α-Fe_2_O_3_ and NiO. In CeO_2,_ oxygen from the gas phase first forms adsorbed superoxide or peroxide species which incorporate into the lattice via the following sequence of reactions [15,16,17]:

$$O_{2\left(g\right)}↔O_{2\left(\mathrm{ads}\right)}↔O_{2\left(\mathrm{ads}\right)}^{-}↔O_{2\left(\mathrm{ads}\right)}^{2-}↔2O_{\left(\mathrm{ads}\right)}^{-}↔2O_{\left(\mathrm{lattice}\right)}^{2-}$$

Isotopic oxygen exchange in gas-solid systems is widely used in heterogeneous catalysis for studying oxygen activation, characterization of its state on the catalyst surface, and elucidation of kinetic peculiarities of oxygen transfer, including bulk oxide oxygen atoms, depending on sample microstructure. The basic theory is based on the classification of exchange types, depending on the number of surface oxygen atoms participating in an exchange with O_2_ (zero-atom, single-atom, or two-atom exchange) [6,18,19,20,21,22]. Regarding the exchange type, one may reveal the most probable mechanisms of oxygen activation on the surface. The unified mechanism was proposed by Muzykantov [14,23,24] for the interpretation of different types of oxygen exchange in oxides. It includes two types of surface oxygen species resulting from oxygen adsorption on the catalyst surface, i.e., O strongly bound with oxygen vacancy (O_v_) and O located on the surface (O_s_). The last, in turn, can diffuse on the surface and occupy the oxygen vacancy, thus transforming to O_v_. Such transformation is reversible: strongly bound oxygen can diffuse to the surface as well. O_2_ formation proceeds by recombination of O_v_ and O_s_. According to Muzykantov’s theory, it is the mobility of O_s_ that determines the type of exchange. Heteroexchange with the participation of two atoms of oxide surface oxygen is realized at a high diffusion rate of O_s_, and one oxygen atom participates in the exchange at a low rate of diffusion. Therefore, one can estimate the mobility of surface oxygen from the ratio of different types of exchange. Boreskov and Muzykantov [14] presented the characteristics of surface oxygen mobility of some simple and complex oxides and noted its high value for α-Fe_2_O_3_. We supposed that the same mechanism of oxygen species recombination could realize in the reaction of N_2_O decomposition.

At high temperatures, the bulk oxide oxygen atoms take part in the exchange process. That is why many recent studies focused on the estimation of the rate of bulk oxygen substitution, which may be used for characterizing the mobility of lattice oxygen [7,25,26]. The oxygen exchange behavior at high temperatures in La-Sr-Mn-O samples has been studied with a special emphasis on its relation to catalytic activity in N_2_O decomposition [7]. It was shown that the appearance of the second faster pathway of oxygen transfer in the bulk of the catalyst through oxygen vacancies or disordered-free channels in the perovskite structure resulted in increased catalytic activity. Evaluation of the rates of surface oxygen exchange and the coefficient of lattice oxygen diffusion in three La-Sr-Fe-O catalysts with close element content but different phases and surface compositions revealed the direct correlation between the surface exchange rate constant and the rate of N_2_O decomposition [27]. The results obtained can be useful for developing active catalysts.

In the present study, we investigated the main features of oxygen transfer on the surface and in the bulk of CeO_2_ and MeO_x_/CeO_2_ (Me = Fe, Co, Ni) samples using ^18^O isotope exchange. It allowed us to reveal the interdependence of these processes and the kinetics of the reaction of N_2_O decomposition.

## 2. Materials and Methods

### 2.1. Samples Preparation and Characterization

Ce, Co, Ni, and Fe nitrates of a purity of 99.0% and citric acid (99.8%) were purchased from Vekton, Saint Petersburg, Russia. Ethylene glycol (99.0%) was purchased from Ecros, Saint Petersburg, Russia.

CeO_2_ (S_BET_ = 1.4 m^2^/g, pore volume 0.42 cm^3^/g) used as the support was obtained using calcination of Ce(NO_3_)_3_·6H_2_O at 900 °C for 36 h. MeO_x_/CeO_2_ samples (Me = Fe, Co, Ni) with Me concentration 6.7 × 10^19^ at/m^2^ (0.86–0.91 wt%) were prepared using incipient wetness impregnation of CeO_2_ with a water solution of a corresponding Me nitrate (0.37 mol/L) with added citric acid (0.41 mol/L) and ethylene glycol (0.26 mol/L). A solution 2.9 times more concentrated (with regard to all components) was taken for preparation of FeO_x_/CeO_2_ with an Fe content of 2.5 wt%. After drying first under an IR lamp at continuous mixing and then—at 150 °C for 6 h, samples were calcined at 900 °C for 4 h. S_BET_ values for FeO_x_/CeO_2_ samples were close to that of CeO_2_ (1.3–1.4 m^2^/g). X-ray powder diffraction (XRD) patterns were recorded using a Bruker D8 diffractometer with Cu Kα monochromatic radiation. Each sample was scanned in the range of 2θ from 10° to 70° with a step 0.05° of 2θ. The surface composition of the samples was investigated using X-ray photoelectron spectroscopy (XPS) using spectrometer SPECS (SPECS, Berlin, Germany) with Al Kα irradiation (hν = 1486.6 eV). The positions of the peaks of Au 4f_7/2_ (84.0 eV) and Cu 2p_3/2_ (932.67 eV) core levels were used for calibration of the binding energy (BE) scale.

### 2.2. Temperature-Programmed Isotopic Exchange (TPIE) of O_2_

Before every experiment, the sample loaded into a reactor (quartz tube, i.d. = 3 mm) was kept in 0.5 vol.% ^16^O_2_ + He flow at 900 °C for 30 min; then, the reactor was cooled down to room temperature in the same flow. At T ≤ 100 °C, this mixture was replaced stepwise by the same one but containing ^18^O_2_ and Ar (1 vol.%) as an inert tracer, and the reactor was heated up to 900 °C (rate of heating 14.7 °C/min). Gas flow rate and catalyst loading were the same in all experiments and amounted to 5.0 L/h and 0.025 g, respectively. Transient changes in the gas isotopic composition (^16^O_2_, ^16^O^18^O, and ^18^O_2_ concentrations) were continuously monitored using a QMS 200 gas analyzer (Stanford Research Systems, Sunnyvale, CA, USA). ^18^O isotope fraction in the gas phase O_2_ (*α(t)*) calculated as *α(t)* = (^16^O^18^O + 2^18^O_2_)/2*∑*O^i^O^j^, where *∑*O^i^O^j^ = ^16^O_2_ + ^16^O^18^O + ^18^O_2_ was a characteristic of oxygen exchange. The general quantity of exchanged oxygen for different samples as related to the mass unit (*N_O_*, at/g) was calculated using the formula

(1) $$N_{O}=N_{A}\frac{2C_{O2}U}{g}\int_{0}^{t}(\alpha^{input}-\alpha )dt$$

where *α^input^* is the isotope fraction in the inlet mixture (0.95), *C_O_*_2_ is inlet O_2_ concentration (0.15 × 10^−2^ mol/mol), *U* is the flow rate of the reaction mixture (mol/s), and *N_A_* is Avogadro number.

### 2.3. Catalytic Activity Measurement

The catalytic activity was measured in a fixed-bed U-shaped reactor (3 mm i.d. quartz tube) loaded with 0.038 g of the sample (particles of 250–500 μm in size) at a flow rate of 18–60 L/h, ambient pressure, and at a temperature range of 600–900 °C. A gas mixture containing 0.15—1.0 vol% N_2_O in He was used. To check out the effect of O_2_, 3 vol.% O_2_ was added to the inlet mixture. Outlet mixture composition was analyzed using a gas chromatograph equipped with Porapack T (i.d. = 3mm, l = 3 m, N_2_O analysis) and NaX (i.d. = 3 mm, l = 2 m, N_2_ analysis) columns. N_2_O conversion (*X_N2O_*) calculated as:

(2) $$X_{N2O}=(C_{N2O}^{0}-C_{N2O})/C_{N2O}^{0}\times 100\%$$

where $C_{N2O}$ and $C_{N2O}^{0}$ —outlet and inlet concentrations of N_2_O were considered a measure of sample activity.

## 3. Results and Discussion

### 3.1. Qualitative Analysis of Oxygen Isotope Exchange Process

**Figure 1:**
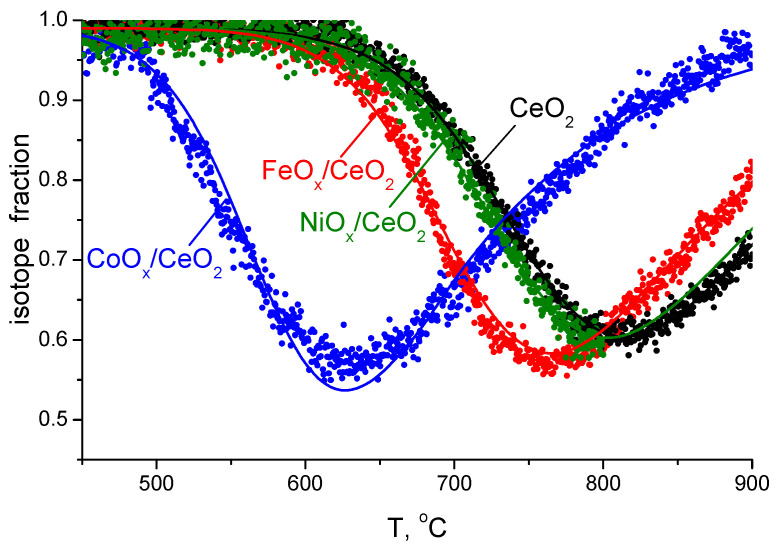
Change of ^18^O fraction in O_2_ during temperature-programmed isotope exchange in different CeO_2_-based catalyst samples. Me = 0.86–0.91 wt.%. Points—experiment, lines—calculation.

The changes in α(t) during the TPIE O_2_ on CeO_2_ and different MeO_x_/CeO_2_ samples depending on T are shown in Figure 1. The ^18^O fraction in O_2_ first decreases as isotope exchange between gas phase oxygen and catalysts starts. Then it goes across the minimum and returns to the initial value after the complete substitution of exchangeable oxygen in the catalyst. One can see that exchange in CoO_x_/CeO_2_ starts at lower temperatures and thus proceeds much faster than in other samples. The quantity of exchangeable oxygen (*N_O_*) in the CoO_x_/CeO_2_ sample is 6.4 × 10^21^ atoms/g (Table 1), which is close to the stoichiometric quantity of O atoms in CeO_2_ lattice (~7 × 10^21^ atoms/g). A lower quantity of oxygen was exchanged in other samples because temperature-programmed heating stopped at 900 °C, while the exchange started at higher temperatures. Based on the similarity of *α(t)* curves for all samples, we supposed that all oxygen of CeO_2_ participates in the exchange therein.

**Figure 2:**
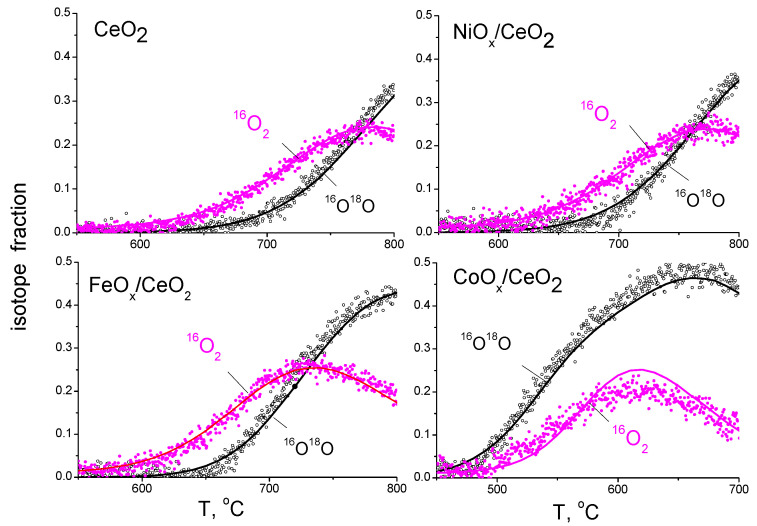
Change of ^16^O_2_ and ^16^O^18^O fractions in O_2_ during TPIE in different samples. Me concentration—0.86–0.91 wt.%. Points—experiment, lines—calculation.

At the same time, a principally different distribution of labeled O_2_ molecules was observed during TPIE (Figure 2). So, in CeO_2_, NiO_x_/CeO_2_, and FeO_x_/CeO_2_ samples, ^16^O_2_ appears in the gas phase first, and its fraction in O_2_ (*f*_32_) exceeds that of ^16^O^18^O (*f*_34_) for a long period of time. In the CoO_x_/CeO_2_ sample, on the contrary, ^16^O^18^O appear first, and *f*_34_ is higher than *f*_32_ for all period of monitoring. Klier and Muzykantov [21,28] proposed the theory of isotope exchange of oxygen with solid oxides, which is currently widely used for the interpretation of mechanisms of oxygen transfer in solids [29,30,31]. With this theory, there are three kinetically distinct types of exchange depending on the number of oxygen atoms from the solid phase participating in the exchange reaction, zero-atom (*R*^0^), single-atom (*R*^I^), and two-atom (*R*^II^). The overall rate of heteroexchange *R* = 0.5*R*^I^ + *R*^II^. Both qualitative and quantitative interdependence between the type of exchange and distribution of isotope molecules during the exchange was determined within the framework of this theory. The observed character of the curves preferentially evidences the two-atom type of exchange in CeO_2_, NiO_x_/CeO_2_, and FeO_x_/CeO_2_ samples where two O atoms of the catalysts participate in every act of exchange:

^18^O_2_ + [^16^O]_cat_ + [^16^O]_cat_ → ^16^O_2_+ [^18^O]_cat_ + [^18^O]_cat_

In turn, only one O atom participates in the exchange in CoO_x_/CeO_2_ sample:

^18^O_2_ + [^16^O]_cat_ → ^16^O^18^O+ [^18^O]_cat_

### 3.2. Quantitative Estimates of Oxygen Isotope Exchange Rates

Numerical analysis of *α_g_* (T), *f*_32_(T), and *f*_34_(T) was performed to make quantitative estimates of the rates of isotope exchange with an account of types of exchange using the model of flow reactor including diffusion of oxygen isotopes in the bulk of the catalysts (see Appendix A). It revealed that oxygen substitution in the bulk of all samples is determined by the rate of “gas phase–surface” exchange. Diffusion of isotopes in the bulk proceeds very fast. Their concentration in bulk is close to that in the surface layer, which evidences the high mobility of the lattice oxygen. Estimated values of the rates of single-atom (*R*^I^) and two-atom (*R*^II^) exchange, as well as overall rates of heteroexchange (*R*) and contributions of two-atom exchange to the overall rate (*R*^II^/*R*), have been presented in Table 1. The accuracy of estimation of the overall rates of heteroexchange is reasonably high, and the calculating error lies within ±5%. The values of the rates of different types of exchange are estimated with lower accuracy (±10%). One can see that *R*, *R*^I^, and *R*^II^ values change in the following order: CeO_2_ ≤ NiO_x_/CeO_2_ < FeO_x_/CeO_2_ < CoO_x_/CeO_2_. *R*^II^ values vary within one order of magnitude, and differences are more substantial for *R* and *R*^I^. *R*^II^/*R* values are as high as 0.9 for CeO_2_, NiO_x_/CeO_2,_ and FeO_x_/CeO_2_ and are only 0.2 for CoO_x_/CeO_2_. Therefore, a substantially higher rate of exchange in the CoO_x_/CeO_2_ sample compared with other CeO2-based samples is due to one-atomic exchange in the former of them.

### 3.3. Comparison of the Rates of N_2_O Decomposition and Oxygen Isotope Exchange

**Figure 3:**
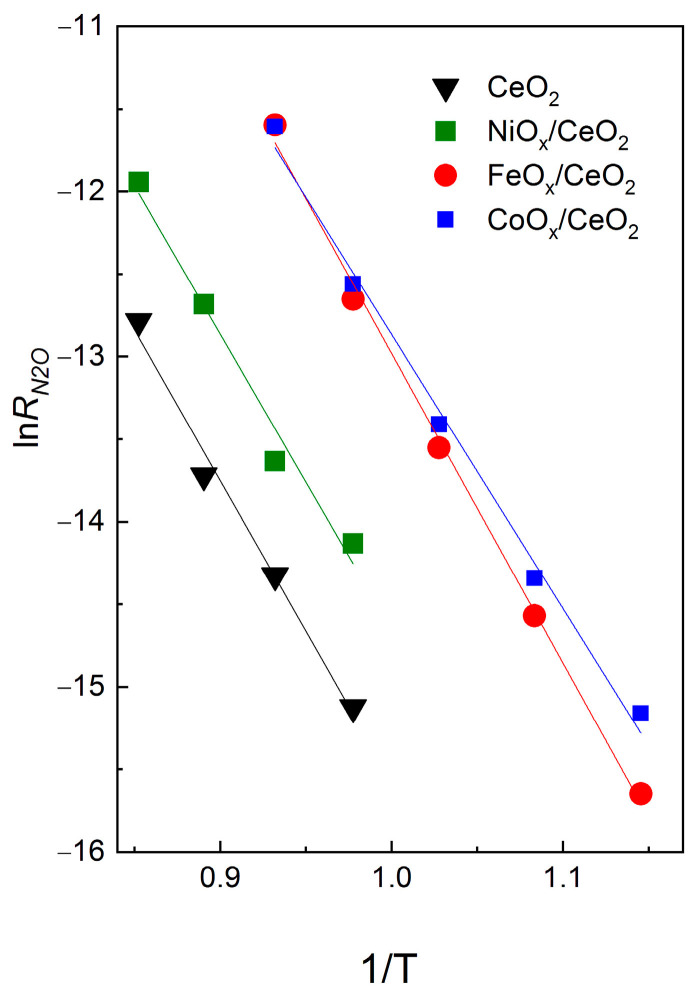
Rates of the reaction of N_2_O decomposition (*R*_N2O_) versus reverse temperature dependencies for different catalysts. Me concentration—0.86–0.91 wt.%. Inlet reaction mixture composition: 0.15%N_2_O in He. T = 600–900 °C.

Dependences of the rates of N_2_O decomposition (*R*_N2O_) versus reverse temperature over different samples have been presented in Figure 3. Their values at 750 °C and activation energy values (*E*_RN2O_), as determined from the Arrhenius dependence on temperature, are presented in Table 2.

One can see that activity in N_2_O decomposition changes in the following order: CeO_2_ < NiO_x_/CeO_2_ < FeO_x_/CeO_2_ < CoO_x_/CeO_2_ excluding the temperature interval above 750 °C where *R*_N2O_ value for FeO_x_/CeO_2_ and CoO_x_/CeO_2_ become close. One can suppose that such approaching activity values at the distinct difference between *R*^I^ and *R*^II^ can be due to the different phase composition of the CoO_x_/CeO_2_ sample under oxygen-deficient conditions (case of deN_2_O) and in the presence of oxygen. Indeed, the reduction of Co_3_O_4_ to CoO took place at above 750 °C during temperature-programmed heating in the inert [8,32,33], while oxygen presence shifted the temperature of the beginning of Co_3_O_4_ reduction to substantially higher temperatures [32,33]. At the same time, CeO_2_, NiO, Fe_2_O_3_, and supported Ni(Fe)O_x_/CeO_2_ samples were stable during TPD in He up to 900 °C, at least [8].

*R*_N2O_ values are substantially lower than *R*^II^ and *R*, but *R*^II^/*R*_N2O_ values for all samples are comparable and lie within 10 (±5), while *R*/*R*_N2O_ values vary from 5 to 70. Therefore, some correlation between *R*_N2O_ and *R*^II^, but not *R*, can be supposed. Below, we attempted to derive steady-state rates kinetic equations using known conceptions about the mechanisms of these reactions. Finally, the *R*^II^/*R*_N2O_ ratios were derived through reaction rate constants and initial concentrations of the reagents.

### 3.4. Mechanism and Form of Rate Equation for Oxygen Isotope Exchange in Oxides

**Scheme 1:**
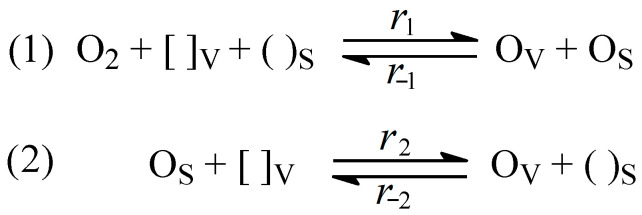
Mechanism of oxygen isotope exchange on the surface of solid oxide.

A certain set of mechanisms can describe every type of oxygen exchange (with the participation of one or two oxygen atoms) in oxides. Single-atom type of exchange is more often described by the Eley–Rideal mechanism that considers the formation of three atomic surface complexes, including two atoms of molecular oxygen and one oxygen atom of the catalyst [34]. The two-atom mechanism is usually interpreted using the two-step mechanism of Bonhoeffer and Farkas [35]. Reversible dissociation of the O_2_ molecule proceeds in the first stage resulting in two equivalent atoms of adsorbed oxygen. In the second stage, the incorporation of adsorbed oxygen to the lattice of oxide proceeds, followed by isotope exchange between adsorbed and lattice oxygens. However, these are the anionic surface defects (oxygen vacancies) that are responsible for the activation of O_2_ on the oxide surface. As a rule, their concentration is low, and it is doubtful whether simultaneous interaction of O_2_ with two defects, which is necessary for molecule dissociation, is possible. We consider that the two-step mechanism proposed by Muzykantov and Boreskov [14,23,24] and presented in Scheme 1 is the most well-grounded.

According to this mechanism, the O_2_ molecule dissociates on oxygen vacancy [ ]_v_ resulting in a strongly bound oxygen atom O_v_ which is included in the oxide lattice, and oxygen atom O_s_ weakly bound with the oxide surface (Scheme 1, stage 1). Formation of O_2_ proceeds by recombination of O_v_ and O_s_. At a high rate of surface diffusion O_s_ can find and occupy another vacant site [ ]_v_ resulting in the formation of O_v_ (Scheme 1, stage 2). This stage is reversible, i.e., strongly bound oxygen can leave this place and come on the surface. As a matter of fact, the second stage characterizes the mobility of O_s_. This mechanism allows the interpretation of both two- and single-atomic exchanges. Two-atom exchange is realized according to the following pathway: step 1 (*r*_1_) → step 2 (*r*_2_) → step −2 (*r*_−2_) → step −1 (*r*_−1_), while the single-atom mechanism includes only two steps: step 1 (*r*_1_) → step −1 (*r*_−1_). The selectivity of the reaction of isotope exchange by pathway corresponding to the two-atom exchange (*S)* is determined by the ratio of the rates of step −1 and step 2:

(3) $$S=\frac{r_{2}}{r_{2}+r_{-1}}=\frac{1}{1+r_{-1}/r_{2}}$$

Muzykantov et al. derived an equation for the rate of two-atomic exchange for the case of its strong limitation by stage 2 (Scheme 1) using this mechanism [14,23,24]. They supposed as well that the degree of the population of [ ]_v_ by strongly bound oxygen (O_v_) is close to 1, while surface coverage by O_s_ → 0. In this case, the order of reaction for O is close to 0.5. In our study, we considered a more general case when the rate-determining step is unknown. No restrictions will be imposed on the concentration of O_v_ as well, and only the degree of surface coverage by O_s_ will be supposed as negligible. In this case, rate equations for stages 1 and 2 (Scheme 1) can be represented as follows:

(4) $$r_{1}=k_{1}C_{O2}(1-\theta_{V})(1-\theta_{S})\approx k_{1}C_{O2}(1-\theta_{V})$$

(5) $$r_{-1}=k_{-1}\theta_{V}\theta_{S}$$

(6) $$r_{2}=k_{2}(1-\theta_{V})\theta_{S}$$

(7) $$r_{-2}=k_{-2}\theta_{V}(1-\theta_{S})\approx k_{-2}\theta_{V}$$

Here, *θ_V_* and *θ_S_* are the concentrations of O_v_ and O_s_, respectively, and *k_i_* are the reaction rate constants of the *i*th stage. Under the conditions of adsorption-desorption equilibrium, the rates of direct and reverse reactions are equal:

(8) $$k_{1}C_{O2}(1-\theta_{V})=k_{-1}\theta_{V}\theta_{S}$$

(9) $$k_{2}\theta_{S}(1-\theta_{V})=k_{-2}\theta_{V}$$

We expressed *θ_v_* and *θ_s_* solving Equations (8) and (9) simultaneously. Finally, substituting them in Equations (4)–(7), we obtained:

(10) $$r_{1}=r_{-1}=\frac{k_{1}C_{O2}}{1+\sqrt{k_{1}C_{O2}k_{2}/k_{-1}k_{-2}}}$$

(11) $$r_{2}=r_{-2}=\frac{\sqrt{k_{1}C_{O2}k_{2}k_{-2}/k_{-1}}}{1+\sqrt{k_{1}C_{O2}k_{2}/k_{-1}k_{-2}}}$$

(12) $$S=\frac{1}{1+r_{-1}/r_{2}}=\frac{1}{1+\sqrt{k_{1}C_{O2}k_{-1}/k_{2}k_{-2}}}$$

The rate of the reaction of two-atomic exchange can be expressed with the following equation:

(13) $$R^{\mathrm{II}}=r_{1}S=\frac{k_{1}C_{O2}}{1+a_{1}\sqrt{k_{1}C_{O2}k_{2}/k_{-1}k_{-2}}+k_{1}C_{O2}/k_{-2}}$$

where

(14) $$a_{1}=1+k_{-1}/k_{2}$$

Detailed derivation of the Equations (10)–(14) is given in Appendix B

In the case $r_{2}>>r_{{}_{-1}}$ (stage 2 is not rate-determining), the following approximate equation is applicable for the description of the two-atomic isotope exchange:

(15) $$R^{\mathrm{II}}\approx \frac{k_{1}C_{O2}}{1+\sqrt{k_{1}C_{O2}k_{2}/k_{-1}k_{-2}}}$$

According to Equation (15), the rate equation can have an order dependence on oxygen concentration (*n*) varying from 0.5 to 1, depending on the value of the $k_{1}C_{O2}k_{2}/k_{-1}k_{-2}$ ratio. In the case of $k_{1}C_{O2}k_{2}>>k_{-1}k_{-2}$ (reaction equilibrium is shifted to direct steps and, thus, *θ_v_* → 1), the *n* value will be close to 0.5. In the opposite case ($k_{1}C_{O2}k_{2}<<k_{-1}k_{-2}$) *n* → 1. According to Equation (13), the *n* value can be even less than 0.5 in the case $k_{1}C_{O2}>k_{-2}$ (the rate of two-atomic exchange is strictly determined by stage 2).

### 3.5. Mechanism and Equation Rate for N_2_O Decomposition Reaction

We observed a decrease in N_2_O conversion in the presence of oxygen in the reaction mixture over FeO_x_/CeO_2_ and CoO_x_/CeO_2_ (Table 3) and FeO_x_/(CeO_2_ + Al_2_O_3_) [9] samples, which can be due to the competitive adsorption of N_2_O and oxygen on the same active sites. Therefore, O_2_ formation by step 3 is preferable for CeO_2_-based samples. However, oxygen species localized in anionic vacancies (O_v_) are strongly bound with cations, and it is doubtful whether they can diffuse easily on the catalyst surface.

**Scheme 2:**
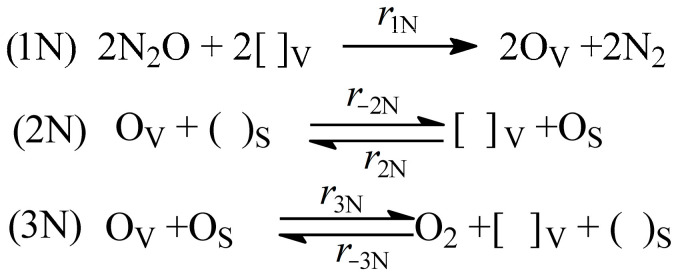
Mechanism of the reaction of N_2_O decomposition.

We supposed that the recombination of two oxygen atoms proceeds with the same mechanism as at oxygen exchange, i.e., with the interaction of O_v_ with O_s_. The last, in turn, is capable of diffusing on the surface and can result from O_v_ (lattice oxygen) coming to the surface. The mechanism of N_2_O decomposition accounting for the above reactions is represented in Scheme 2.

We consider that oxygen re-adsorption by reverse stage 3N (Scheme 2) is negligible because of low oxygen concentration at low N_2_O conversion values in the experiments done for reaction rate evaluation and fast oxygen washing out from the reactor. In this case, the degree of surface coverage with O_s_ under reaction conditions can be supposed as negligible, like in the reaction of oxygen isotope exchange. One can see that direct and reverse stages 2N and 3N (Scheme 2) are represented in the scheme of oxygen isotope exchange (Scheme 1) as well. Therefore, the rates of these stages can be expressed in terms of rate constants of isotope exchange as follows:

(16) $$r_{2N}=k_{2}(1-\theta_{V})\theta_{S}$$

(17) $$r_{-2N}=k_{-2}\theta_{V}$$

(18) $$r_{3N}=k_{-1}\theta_{V}\theta_{S}$$

Designating the rate constant of the stage of N_2_O adsorption as *k_1N_,* one can obtain the equation for *r_1N_*:

(19) $$r_{1N}=k_{1N}C_{N2O}(1-\theta_{V})$$

Under the steady state: $r_{1N}=r_{-2N}-r_{2N}=r_{3N}$, so the system of steady-state equations looks as follows:

(20) $$2k_{1}{}_{N}C_{N2O}\times (1-\theta_{V})=k_{-1}\times \theta_{V}\times \theta_{S}$$

(21) $$k_{-2}\times \theta_{V}=k_{-1}\times \theta_{V}\times \theta_{S}+k_{2}\times (1-\theta_{V})\times \theta_{S}$$

Solving Equations (20) and (21) simultaneously, we found *θ_V_* and *θ_S_*. Finally, substituting them in the expression for *r_1N_*, we obtained the following kinetic equation for the rate of reaction of N_2_O decomposition (see details in Appendix C):

(22) $$R_{N2O}=2k_{1N}C_{N2O}(1-\theta_{V})=\frac{2k_{1N}C_{N2O}}{1+a_{2}\sqrt{2k_{1N}C_{N2O}k_{2}/k_{-1}k_{-2}}+k_{1}{}_{N}C_{N2O}/k_{-2}}$$

where

(23) $$a_{2}=\sqrt{1+k_{-1}/k_{2}}$$

The following approximate kinetic equation is applicable in the case $k_{1}{}_{N}C_{N2O}/k_{-2}$ (stage 2N is not rate-determining):

(24) $$R_{N2O}=2k_{1N}C_{N2O}(1-\theta_{V})\approx \frac{2k_{1N}C_{N2O}}{1+\sqrt{2k_{1N}C_{N2O}k_{2}/k_{-1}k_{-2}}}$$

One can see that the equations for *R_N2O_*, Equation (22) and *R^II^* Equation (13), are close by the form. In the general case, the ratio between the rates of these reactions is determined with the following equation:

(25) $$\frac{R^{II}}{R_{N2O}}=\frac{k_{1}C_{O2}}{2k_{1N}C_{N2O}}\frac{1+a_{2}\sqrt{2k_{1N}C_{N2O}k_{2}/k_{-1}k_{-2}}+k_{1}{}_{N}C_{N2O}/k_{-2}}{1+a_{1}\sqrt{k_{1}C_{O2}k_{2}/k_{-1}k_{-2}}+k_{1}C_{O2}/k_{-2}}$$

In the case when stages 2 (Scheme 1) and 2N (Scheme 2) are not rate-determining for both reactions, the ratio between rates of these reactions is determined with the following approximate equation:

(26) $$\frac{R^{II}}{R_{N2O}}\approx \frac{k_{1}C_{O2}}{2k_{1N}C_{N2O}}\frac{1+\sqrt{2k_{1N}C_{N2O}k_{2}/k_{-1}k_{-2}}}{1+\sqrt{k_{1}C_{O2}k_{2}/k_{-1}k_{-2}}}$$

According to literature data, on oxides, the rate is usually proportional to C_N2O_ or has a slightly lower order due to the inhibition of produced oxygen [36]. In line with this, the dependence of the rate on N_2_O concentration (varied from 0.15 vol.% to 1.0 vol.%) was around 0.97 for FeO_x_/CeO_2_ (Appendix D). Such close to 1 order means that the value of $\sqrt{2k_{1N}C_{N2O}k_{2}/k_{-1}k_{-2}}$ in Equations (24) and (26) is much less than unity. In this case, the ratio between the rates of reactions of two-atomic exchange and N_2_O decomposition depends on the values of rate constants of the stages of oxygen and N_2_O adsorption and their concentrations.

### 3.6. Mobility of Surface Oxygen and Rate Determining Steps of Two Atomic Isotope Exchange and N_2_O Decomposition Reaction

According to the proposed mechanisms of isotope exchange and N_2_O decomposition, the mobility of surface oxygen is characterized by the rate of the same stage designated as stage 2 (Scheme 1) and 2N (Scheme 2). One can estimate the rate of this stage in the reaction of isotope exchange directly from experimental data. So, at known ratios of *R*^I^ and *R*^II^ (Table 1) to the overall rate of oxygen adsorption-desorption, one can estimate the selectivity of exchange running by different pathways. The rate of oxygen adsorption-desorption is equal to the sum of *R*^I^ and *R*^II^:

(27) $$r_{-1}=r_{1}=R^{\mathrm{II}}+R^{I}$$

Therefore, the selectivity of the reaction of isotope exchange with the pathway corresponding to the two-atomic exchange (*S*) will be determined in the following way:

(28) $$S=\frac{R^{\mathrm{II}}}{r_{-1}}=\frac{R^{\mathrm{II}}}{R^{\mathrm{II}}+R^{I}}$$

At a known $r_{-1}$ and *S*, one can calculate the rate of stage 2 (Scheme 1) using Equation (3):

(29) $$r_{2}=r_{-1}S/(1-S)$$

Calculated values of *S* and the rates of different stages of isotope exchange in the samples have been presented in Table 4. The value of *S* for CoO_x_/CeO_2_ is low, and the estimation of *r_2_ = r_−2_,* in this case, is quite precise. The higher the selectivity, the less precise the estimate.

The values of *θ_V_* and *θ_S_* in the reaction of N_2_O decomposition can differ from those in isotope exchange, thus resulting in the change of the ratio between rates of these stages. So, the *r*_−2_/*r*_−1_ ratio in the reaction of isotope exchange is determined with the values corresponding rate constants and *θ_S_* value:

(30) $$\frac{r_{-2}}{r_{-1}}=\frac{k_{-2}\theta_{V}}{k_{-1}\theta_{V}\theta_{S}}=\frac{k_{-2}}{k_{-1}\theta_{S}}$$

The ratio *r_−2N_/r_3N_* that determines the rate-determining stage in the reaction of N_2_O decomposition is the same:

(31) $$\frac{r_{-2N}}{r_{3N}}=\frac{k_{-2}\theta_{V}}{k_{-1}\theta_{V}\theta_{S}}=\frac{k_{-2}}{k_{-1}\theta_{S}}$$

The formation of weakly bound oxygen O_s_ proceeds with both stage 2N and stage of oxygen adsorption 3N (Scheme 2). Gas phase oxygen concentration under the conditions of N_2_O decomposition (<0.01% on average by the length of the catalyst layer) is much less than during isotope exchange experiments (0.5%). Accordingly, we consider that the *θ_S_* value in the reaction conditions cannot be higher than at isotope exchange. In this case

(32) $$\frac{r_{-2N}}{r_{3N}}\geq \frac{r_{-2}}{r_{-1}}$$

$r_{-2}$ >> $r_{-1}$ for CeO_2_, NiO_x_/CeO_2_, and FeO_x_/CeO_2_ samples. Therefore $r_{-2N}$ >> $r_{3N}$ as well. In this case, stages 2 (Scheme 1) and 2N (Scheme 2) are not rate-determining in the reaction of oxygen isotope exchange and in N_2_O decomposition. The ratio between the rates of these reactions is thus determined with Equation (26) and depends mainly on the ratio of rate constants of the stages of oxygen and N_2_O adsorption and their concentrations.

In the case of CoO_x_/CeO_2_, $r_{-2}$ < $r_{-1}$, i.e., stage 2 is rate-determining in the reaction of isotope exchange. However, it is not so obvious for the reaction of N_2_O decomposition because $r_{-2N}$/$r_{3N}$ can be higher than $r_{-2}$/$r_{-1}$. Finally, the ratio between rates of these reactions will be determined with Equation (25).

### 3.7. Role of MeO_x_–CeO_2_ Interface in the Reactions of Isotope Exchange and N_2_O Decomposition

**Figure 4:**
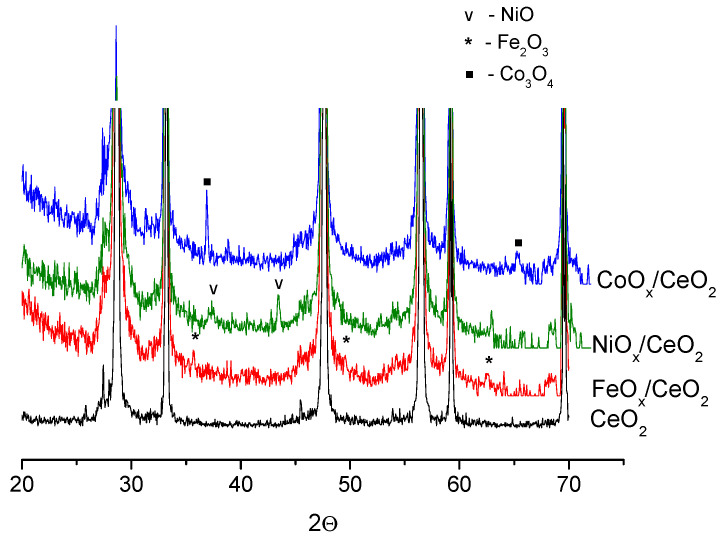
XRD patterns of MeO_x_/CeO_2_ (Me = Fe, Co, Ni) samples. Me concentration—0.86–0.91 wt.%.

Estimates of the rates of different stages made above were based on the assumption of equivalence of all active sites participating in the reactions. It is true only for CeO_2_. Supporting of MeOx produces other sites which can be responsible for efficient O_2_ adsorption as well. First of all, these are oxygen vacancies located on the surface of crystalline Fe_2_O_3_, NiO, and Co_3_O_4_ particles (Figure 4) detected in the samples or their smaller clusters and on the Me–Ce interface. The relative surface concentration of MeO_x_ is reasonably low (Me/Ce = 0.13 ± 0.1) (Table 5), but their contribution to the rate of exchange can be prominent.

We estimated the values of R^I^ and R^II^ on MeO_x_ normalized to their concentration (*R*^I^ _(Me,Ce)_ and *R*^II^ _(Me,Ce)_, respectively) using Me/Ce values on the surface as measured with XPS and supposing overall concentration of active sites as 1.67 × 10^5^ mole/m^2^. Then *S* values and rates of reactions of stages 1 and 2 (Scheme 1) for isotope exchange on different sites ((*r*_1_ = *r*_−1_) _(Me,Ce)_ and (*r*_2_ = *r*_−2_)(_Me,Ce)_) were calculated as well using Equations (27)–(29) (Table 5).

**Scheme 3:**
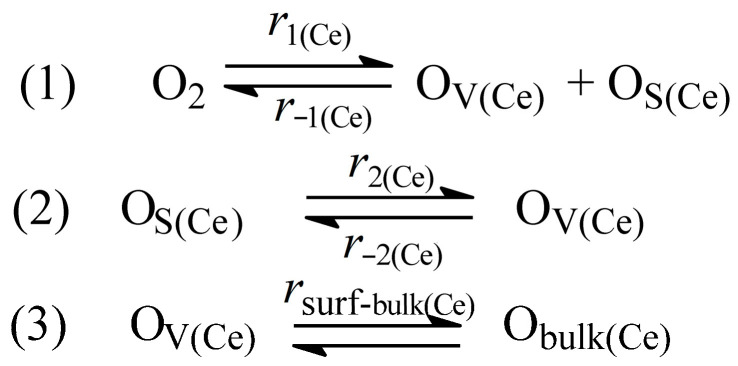
Mechanism of isotope transfer in CeO_2_.

It is logical to suppose that exchange between surface and bulk oxygen on oxides proceeds with the participation of strongly bound oxygen O_V_. Scheme 3 describes isotope transfer between gas phase O_2_ and ceria bulk (O_bulk(Ce)_) for this case.

**Scheme 4:**
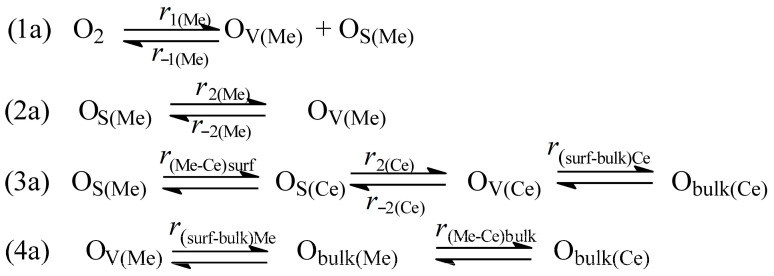
Mechanism of isotope transfer in MeO_x_/CeO_2_ samples.

To keep the general rate of heteroexchange and selectivity to the two-atom exchange observed in the experiments, the rate of stage 3 (Scheme 3) should be at least equal to the sum of the rates of stages 1 and 2, i.e., *r*_surf_*-*_bulk(Ce)_ ≥ *r*_1(Ce)_ + *r*_2(Ce)_. An additional pathway of isotope transfer between O_2_ and ceria bulk appears in MeO_x_/CeO_2_ samples with the participation of MeO_x_ particles (Scheme 4).

This scheme includes two pathways of isotope transfer from the MeO_x_ particle to O_bulk(Ce)_. Spillover of weakly bound oxygen from the MeO_x_ surface to the CeO_2_ surface followed by transfer to CeO_2_ bulk through O_v(Ce)_ (stage 3a, Scheme 4) provides for the first pathway. By the second pathway (stage 4a), the exchange proceeds first—between strongly bound oxygen of MeO_x_ (O_V(Me)_) and that in MeO_x_ bulk (O_bulk(Me)_)_,_ and then—between O_bulk(Me)_ and oxygen in CeO_2_ bulk, Ob_ulk(Ce)_. The overall rate of isotope transfer by stages 3a and 4a cannot be less than *r*_1(Me)_ + *r*_2(Me)_.

Numerical analysis of isotope experiments showed that isotope diffusion to the bulk of CeO_2_ (O_s(Ce)_ ↔ O_bulk(Ce)_) is not the rate-determining step of the isotope exchange reaction. This agrees with the results of earlier studies performed on ceria [37], where higher mobility of the oxygen in the lattice than the rate of exchange with the gas phase has been shown. In contrast with CeO_2_, the rate of isotope exchange of the surface and the bulk oxygen in Fe, Co, Ni, or mixed La-Fe-O oxides is much lower than the rate of exchange with gas phase O_2_ [37,38,39]. So, in the LaFeO_3_ sample representing a mixture of perovskite, α-Fe_2_O_3,_ and La_2_O_3,_ the rate constant of heteroexchange at 800 °C was 8 s^−1^, while the rate of oxygen diffusion in bulk was three orders of magnitude lower (6 × 10^−3^ s^−1^). In CeO_2_, vice versa, these values were 1.2 s^−1^ and >0.1 s^−1^, respectively [39]. Solsona et al. performed ^18^O_2_ TPIE experiments and showed that the addition of ceria into the NiO catalysts increases the diffusion of oxygen in the bulk of the samples [40]. Hence, we suppose that the rate of stage 4a in all MeO_x_/CeO_2_ samples is negligible compared with the rate of oxygen exchange on the surface of the MeO_x_ particle.

We tried to estimate whether the rate of stage 3a (Scheme 4) can provide an increase in the rate of heteroexchange in MeO_x_/CeO_2_ compared with CeO_2_. At the known Me/Ce value (Table 5), the rate of transfer of isotope label by stage 3a (Scheme 4) will be not higher than *r*_2(Ce)_/0.11 = 42 (±20) mole/(mole(Me) × s) for any of MeO_x_/CeO_2_ samples. For the case of FeO_x_/CeO_2_ and CoO_x_/CeO_2,_ this value is less than *r*_1(Me)_ + *r*_2(Me)_ (Table 5). Therefore, one can conclude that the increase of the rate of isotope exchange in FeO_x_/CeO_2_ and CoO_x_/CeO_2_ samples compared with that in CeO_2_ is due to sites on the Me–CeO_2_ interface characterized by increased concentration of oxygen vacancies promoting oxygen exchange [41,42,43].

**Figure 5:**
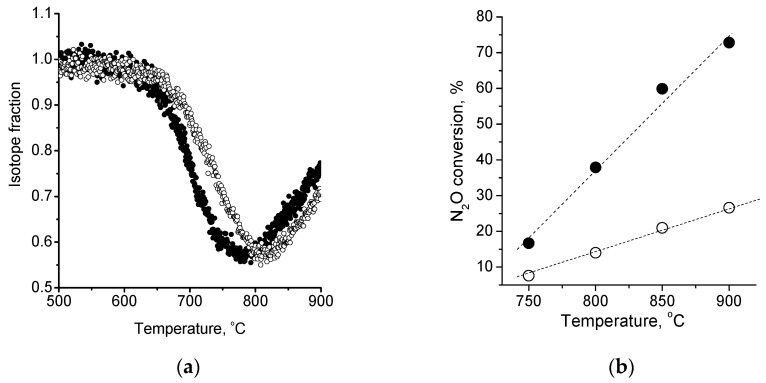
Oxygen isotope fraction during ^18^O_2_ TPIE (**a**) and N_2_O conversion variation with temperature (**b**) for FeO_x_/CeO_2_ samples with different Fe content (•—0.86 wt.% Fe, o—2.5 wt.% Fe). Inlet mixture composition: 0.15% N_2_O in He, 60 L/h.

For the case of FeO_x_/CeO_2,_ this is confirmed with ^18^O_2_ TPIE experiments on the samples with different Fe content. One can see (Figure 5a) that the increase of Fe content in the sample from 0.86 wt% to 2.5 wt% resulted in a high-temperature shift in the isotope fraction curve, thus evidencing a decrease in the rate of exchange. Non-proportional growth of the Fe/Ce surface ratio (as measured with XPS) from 0.13 to 0.19 only with Fe content in the sample can be due to the enlargement of Fe_2_O_3_ particles decreasing the concentration of sites on the Fe–Ce interface. It is interesting that FeO_x_/CeO_2_ sample with higher Fe content was also less active in deN_2_O (Figure 5b). Therefore, reaction on the Me–CeO_2_ interface contributes more substantially to the overall reaction rate than that on Fe sites.

The results of TG-DTA-MS experiments performed in an oxygen-containing stream [33] showed that cobalt in CoOx–CeO_2_ catalysts are present in the form of Co_3_O_4_/CoO particles and a CoO_x_–CeO_2_ system formed on the cobalt oxide–ceria interface. The decrease of N_2_O conversion under oxygen-deficient conditions at T > 800 °C resulted from the reduction of Co_3_O_4_ to CoO in the particles weakly interacting with CeO_2_. The authors, therefore, concluded that the activity of these catalysts originates from the interaction of cobalt oxide with CeO_2_. A strong dispersion effect of the cobalt spinel active phase spread over ceria on the turnover rate of deN_2_O was observed due to the progressive agglomeration of the Co_3_O_4_ nanocrystallites into compact domains with the increasing cobalt loading [44]. Model supposing the cylindrical shape of Co_3_O_4_ domains permitted normalizing the deN_2_O reaction rate with respect to the cobalt content and the length of the Co_3_O_4_/CeO_2_ interface periphery. The authors proposed a two-step mechanism operating at the interface, where the redox properties of the cobalt component were responsible for the dissociation of N_2_O molecules and formation of oxygen intermediates, whereas the ceria periphery was responsible for the enhanced diffusion and recombination of oxygen adspecies, closing the catalytic cycle.

For the NiO_x_/CeO_2_ sample, we did not observe the effect of the Ni-ceria interface on the rate of oxygen transfer. Indeed, a minimal red shift of the CeO_2_ absorption edge was observed in the UV-vis DR spectra of the NiO_x_/CeO_2_ sample compared to that in the CeO_2_ (Eg value of 3.35 eV and 3.27 eV, respectively). For the CoO_x_/CeO_2_ and FeO_x_/CeO_2_ samples, Eg values were substantially lower (3.18–3.22 eV) [8]. It indicates that either (1) Fe/Co ions substitute Ce into the CeO_2_ lattice, or (2) the isolated Me ions form a mixed Me–Ce oxide and strongly interact with CeO_2_ leading to the electronic structure changes [45]. The additional absorption band at ~20,000–23,500 cm^−1^ in the spectra of Co(Fe)O_x_/CeO_2_ samples [8] is due to O_2_ adsorption on the structural defect; more obviously, clusters of oxygen vacancies arose on Fe(Co)-CeO_2_ interface.

## 4. Conclusions

Oxygen transfer on the surface and in the bulk of CeO_2_ and MeO_x_/CeO_2_ (Me = Fe, Co, Ni) catalysts was studied using ^18^O isotope exchange. The relationship between this process and the kinetics of the reaction of N_2_O decomposition was elucidated.

Supporting of MeO_x_ onto CeO_2_ increased the rate of isotope exchange by 20% (Ni), threefold (Fe), and more than ten times (Co). Both single-atom and two-atom exchanges proceed simultaneously in all samples: two-atom exchange predominates in CeO_2_, NiO_x_/CeO_2,_ and FeO_x_/CeO_2_, while the one-atom exchange prevails in CoO_x_/CeO_2_. One can interpret both types of exchange using a two-step mechanism [14,18,19], considering two forms of surface oxygen. With this mechanism, weakly bound oxygen (O_s_) can diffuse on the surface and can transform into a strongly bound form (O_v_) after interaction with anionic vacancy. The contribution of every type of exchange to the overall rate of heteroexchange depends on the ratio of the rate of recombination of O_s_ and O_v_ and the rate of their interconversion. The last, in turn, characterizes the mobility of the surface oxygen. We obtained estimates of the surface oxygen mobility in CeO2-based samples using such a model. It turned out that CeO_2_ promotion using FeO_x_ and NiO_x_ increases the mobility of surface oxygen, while it doesn’t change in the case of CoO_x_. It is shown that the mobility of surface oxygen can determine the rate of the reaction of N_2_O decomposition on CoO_x_/CeO_2_. On other samples, it is determined by the rate of N_2_O adsorption.

Detailed analysis of the kinetics of isotope exchange accounting for the surface concentration of MeO_x_ made it possible to estimate their contribution to the rates of different types of exchange and quantify the rates of oxygen transfer from MeOx to the bulk of CeO_2_ by different pathways. Oxygen transfer from MeOx to the bulk of CeO_2_ realized on the Me–Ce interface is shown to be important in FeOx/CeO_2_ and CoOx/CeO_2_ and makes a prominent contribution to the overall rate of exchange in these samples. Direct dependence of the rate of isotope exchange in FeO_x_/CeO_2_ samples with different Fe content on the length of the FeO_x_-CeO_2_ interface was shown. The analogous effect was observed for the rate of deN_2_O reaction, which points to the principal role of the interface in both reactions.

## Figures and Tables

**Table 1:** Quantities of exchangeable oxygen during TPIE (*N_O_*, 1 × 10^21^ at/g), rate values *R*, *R*^I^, *R*^II^ (mole/m^2^s) for different types of oxygen exchange at reference temperature 750 °C and their activation energies *E_R_* (kJ/mole) for different samples.

Sample	*N_O_*	*R* × 10^5^	*R*^I^ × 10^5^	*R*^II^ × 10^5^	*R*^II^/*R*	*E_R_*
CeO_2_	4.5 ^1^	0.42 (±0.02)	0.03 (±0.01)	0.4 (±0.02)	0.95 (±0.05)	170 (±10)
NiO_x_/CeO_2_	2.2 ^2^	0.52 (±0.02)	0.03 (±0.01)	0.5 (±0.02)	0.96 (±0.55)	170 (±10)
FeO_x_/CeO_2_	5.4 ^1^	1.4 (±0.08)	0.1 (±0.02)	1.3 (±0.08)	0.93 (±0.04)	175 (±10)
CoO_x_/CeO_2_	6.4	26 (±1)	41 (±1)	5.5 (±0.5)	0.21 (±0.02)	160 (±10)

^1^ not complete exchange (see Figure 1). ^2^ not complete exchange (heating up to 820 °C only, afterward, the constant temperature was kept, see Figure 1).

**Table 2:** Rates of the reaction of N_2_O decomposition (*R*_N2O_) and oxygen isotope exchange (*R*, *R*^II^) at 750 °C. Inlet reaction mixture compositions: 0.15% N_2_O in He and 0.5% O_2_ in He, respectively.

Sample	*R*_N2O_ × 10^6^, mol/m^2^s	*E*_RN2O,_ kJ/mole	*R*^II^/*R*_N2O_	*R*/*R*_N2O_
CeO_2_	0.27	152	15	16
NiO_x_/CeO_2_	0.73	153	7	7
FeO_x_/CeO_2_	3.2	156	5	5
CoO_x_/CeO_2_	3.7	138	14	70

**Table 3:** Effect of oxygen presence in the 0.15% N_2_O in He mixture on N_2_O conversion over CeO_2_-based samples. 900 °C.

Sample	N_2_O Conversion, %	
	0.15% N_2_O in He	0.15% N_2_O + 3% O_2_ in He
FeO_x_/CeO_2_	72.8	63.8
CoO_x_/CeO_2_	70.8	65.4

**Table 4:** Selectivity to two-atom exchange (*S*) and rates of different stages of oxygen isotope exchange.

Sample	*S*	(*r*_1_ = *r*_−1_) × 10^5^ mol/m^2^s	(*r*_2_ = *r*_−2_) × 10^5^ mol/m^2^s	*r*_2(−2)_/*r*_1(−1)_
CeO_2_	0.93 (±0.04)	0.43 (±0.03)	8 (±3)	20 (±10)
NiO_x_/CeO_2_	0.94 (±0.04)	0.53 (±0.03)	15 (±7)	30 (±20)
FeO_x_/CeO_2_	0.92 (±0.04)	1.4 (±0.1)	16 (±5)	12 (±5)
CoO_x_/CeO_2_	0.12 (±0.01)	46 (±1.5)	7 (±1)	0.15

**Table 5:** Me/Ce (Me = Ni, Fe, Co) atomic ratios in the surface layers (XPS) of different MeO_x_/CeO_2_ samples, normalized rates of isotope exchange on MeOx and CeO_2_ (*R*^I^ _(Me,Ce)_ and *R*^II^ _(Me,Ce)_) and rates of reactions of stages 1 and 2 (Scheme 1) of isotope exchange on different sites ((*r*_1_ = *r*_−1_) _(Me,Ce)_ and (*r*_2_ = *r*_−2_) _(Me,Ce)_). Me concentration—0.86–0.91 wt.%.

Sample	Me/Ce	*R*^I^ _(Me,Ce),_ s^−1^	*R*^II^ _(Me,Ce),_ s^−1^	(*r*_1_ = *r*_−1_) _(Me,Ce)_, s^−1^	(*r*_2_ = *r*_−2_) _(Me,Ce)_, s^−1^
CeO_2_		0.015	0.245	0.26 (±0.02)	4.8 (±2)
NiO_x_/CeO_2_	0.12	0.04	0.68	0.72 (±0.02)	12 (±6)
FeO_x_/CeO_2_	0.13	0.4	4.3	4.7 (±0.05)	48 (±15)
CoO_x_/CeO_2_	0.14	190	23	210 (±7)	25 (±3)

**Figure A1:**
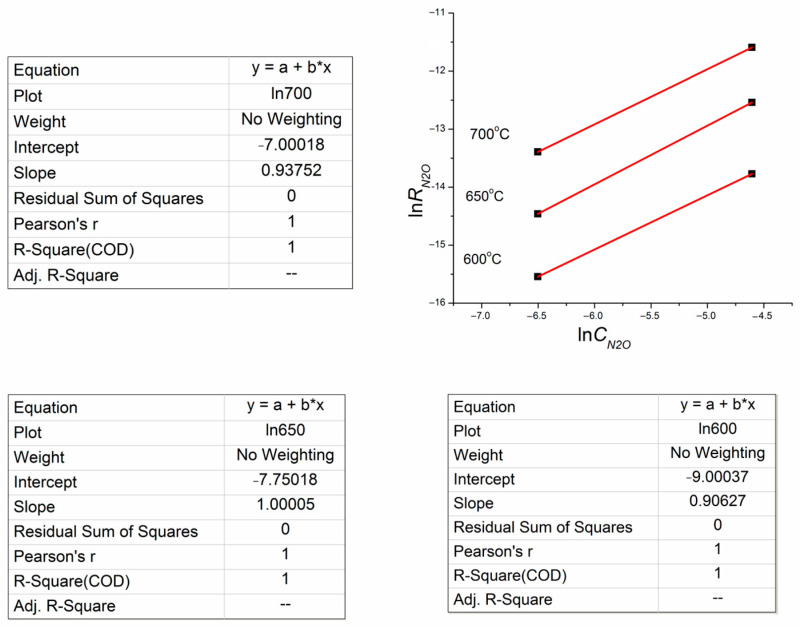
Dependence of the logarithm of the reaction rate on the logarithm of N_2_O concentration (% vol) at different temperatures.

## Appendix A. Numerical Analysis of Temperature Programmed Isotope Exchange of Oxygen

The model of isotope exchange in the plug flow reactor represents the system of hyperbolic and ordinary differential equations:

(A1) $$\beta \frac{\partial \alpha }{\partial T}+\frac{1}{\tau }\frac{\partial \alpha }{\partial \xi }=b(0.5R^{I}+R^{\mathrm{II}})(\alpha_{OX}-\alpha )$$

(A2) $$\beta \frac{\partial \alpha_{s}}{\partial T}=(0.5R^{1}+R^{2})(\alpha_{g}-\alpha_{s})-\frac{N_{bulk}}{N_{S}}{\frac{D}{h^{2}}\frac{\partial \alpha_{bulk}}{\partial \eta }|}_{\eta =1}$$

(A3) $$\beta \frac{\partial \alpha_{Obulk}}{\partial T}=\frac{D}{h^{2}}\frac{\partial^{2}\alpha_{Obulk}}{\partial \eta^{2}}$$

(A4) $$\begin{array}{l} \beta \frac{\partial f_{34}}{\partial T}+\frac{1}{\tau }\frac{\partial f_{34}}{\partial \xi }=b[R^{I}(\alpha (1-\alpha_{OX})+\alpha_{OX}(1-\alpha )-f_{34(I)})+\\ R^{\mathrm{II}}(2\alpha_{OX}(1-\alpha_{OX})-f_{34(\mathrm{II})})] \end{array}$$

$$R^{\left(i\right)}=R_{ref}^{\left(i\right)}\exp(-E/RT^{*}),\;D^{\left(i\right)}=D_{ref}^{\left(i\right)}\exp(-E/RT^{*}),\;T^{*}=\frac{TTref}{Tref-T}$$

Initial and boundary conditions:

$$T=T_{0}:\;\alpha =\alpha_{OX}=f_{34}=0$$

$$\xi =0:\;\alpha =\alpha^{input}$$

$$\eta =1:\;\alpha_{bulk}=\alpha_{s},\;\eta =0:\;\frac{\partial \alpha_{bulk}}{\partial \eta }=0$$

Here, α is an atomic fraction of ^18^O in O_2_, *f*_34_ is a fraction of ^16^O^18^O in O_2_, *β* is the rate of heating, τ is the residence time (s), and ξ is the dimensionless reactor length. The atomic fraction of ^16^O_2_ ($f_{32}$) is expressed in terms of α and $f_{34}$ as $f_{32}=1-\alpha -0.5f_{34}$.

Values τ and b in the plug flow reactor can be determined as follows:

$$\tau =\frac{V_{R}}{U},\;b=\frac{N_{OX}}{N_{G}}=\frac{L_{OX}W}{2C_{O_{2}}N_{A}V_{R}}$$

where *U* is the flow rate [cm^3^/s], *V* is the gas phase volume [cm^3^], *L* is the number of oxygen atoms per gram of catalyst [mol/g], W is the catalyst weight [g], $C_{O_{2}}$ is the gas phase oxygen concentration (mol/mol), *N_A_* is the number of gas molecules per unit volume [mol/cm^3^].

The system of Equations (A1)–(A4) was solved numerically. ξ derivatives were replaced with difference quotients of second-order approximation. A derived system of ordinary differential equations (ODE) was solved using the fifth-order Runge-Kutta-Merson method. Calculations with 2- and 10-fold decreased increments of ξ were used to estimate the accuracy of the solution and check out the stability of the system.

## Appendix B. Derivation of an Equation for the Rate Oxygen Two-Atom Exchange

The system of equations of material balance of oxygen under the conditions of adsorption-desorption equilibrium is written as follows:

(A5) $$k_{1}C_{O2}(1-\theta_{V})=k_{-1}\theta_{V}\theta_{S}$$

(A6) $$k_{2}\theta_{S}(1-\theta_{V})=k_{-2}\theta_{V}$$

One can express *θ_S_* from Equation (A6):

(A7) $$\theta_{S}=\frac{k_{-2}\theta_{V}}{k_{2}(1-\theta_{V})}$$

and substitute it for Equation (A5):

(A8) $$k_{1}C_{O2}(1-\theta_{V})=k_{-1}\theta_{V}\frac{k_{-2}\theta_{V}}{k_{2}(1-\theta_{V})}$$

or

(A9) $$\frac{\theta_{V}}{1-\theta_{V}}=\sqrt{k_{1}C_{O2}k_{2}/k_{-1}k_{-2}}$$

One can express *θ_V_* and (1 − *θ_V_*) from Equation (A9):

(A10) $$\theta_{V}=\frac{\sqrt{k_{1}C_{O2}k_{2}/k_{-1}k_{-2}}}{1+\sqrt{k_{1}C_{O2}k_{2}/k_{-1}k_{-2}}}$$

(A11) $$1-\theta_{V}=\frac{1}{1+\sqrt{k_{1}C_{O2}k_{2}/k_{-1}k_{-2}}}$$

and substitute them to Equation (A7), resulting in:

(A12) $$\theta_{S}=\frac{k_{-2}\theta_{V}}{k_{2}(1-\theta_{V})}=\sqrt{k_{1}C_{O2}k_{-2}/k_{-1}k_{2}}$$

Substitution of Equations (A10)–(A12) in the equations of the rates of steps 1 and 2 results in:

(A13) $$r_{1}=r_{-1}=\frac{k_{1}C_{O2}}{1+\sqrt{k_{1}C_{O2}k_{2}/k_{-1}k_{-2}}}$$

(A14) $$r_{2}=r_{-2}=\frac{k_{-2}\sqrt{k_{1}C_{O2}k_{2}/k_{-1}k_{-2}}}{1+\sqrt{k_{1}C_{O2}k_{2}/k_{-1}k_{-2}}}$$

(A15) $$r_{1}/r_{2}=\frac{k_{1}C_{O2}}{k_{-2}\sqrt{k_{1}C_{O2}k_{2}/k_{-1}k_{-2}}}=\sqrt{k_{1}C_{O2}k_{-1}/k_{2}k_{-2}}$$

In this case selectivity of two-atom exchange can be represented using the following equation:

(A16) $$S=\frac{1}{1+r_{1}/r_{2}}=\frac{1}{1+\sqrt{k_{1}C_{O2}k_{-1}/k_{2}k_{-2}}}$$

As a result, we obtained the following equation describing the rate of two-atom exchange:

(A17) $$\begin{array}{l} R^{\mathrm{II}}=r_{1}S=\frac{k_{1}C_{O2}}{\left(1+\sqrt{k_{1}C_{O2}k_{2}/k_{-1}k_{-2}})(1+\sqrt{k_{1}C_{O2}k_{-1}/k_{2}k_{-2}}\right)}=\\ \frac{k_{1}C_{O2}}{1+(1+k_{-1}/k_{2})\sqrt{k_{1}C_{O2}k_{2}/k_{-1}k_{-2}}+k_{1}C_{O2}/k_{-2}} \end{array}$$

Provided $r_{1}/r_{2}=\sqrt{k_{1}C_{O2}k_{-1}/k_{2}k_{-2}}$ << 1, i.e., *r*_1_ is rate-determining (*S* ≈ 1), then:

(A18) $$R^{\mathrm{II}}\approx r_{1}=\frac{k_{1}C_{O2}}{1+\sqrt{k_{1}C_{O2}k_{2}/k_{-1}k_{-2}}}$$

## Appendix C. Derivation of an Equation for Steady State Rate of N_2_O Decomposition

The system of equations of material balance under steady-state conditions of N_2_O decomposition reaction can be written as follows:

(A19) $$2k_{1}{}_{N}C_{N2O}(1-\theta_{V})=k_{-1}\theta_{V}\theta_{S}$$

(A20) $$k_{-2}\theta_{V}=k_{-1}\theta_{V}\theta_{S}+k_{2}(1-\theta_{V})\theta_{S}$$

One can express *θ_S_* from Equation (A20):

(A21) $$\theta_{S}=\frac{k_{-2}\theta_{V}}{k_{-1}\theta_{V}+k_{2}(1-\theta_{V})}$$

and substitute it for Equation (A19):

(A22) $$2k_{1}{}_{N}C_{N2O}(1-\theta_{V})=k_{-1}\theta_{V}\frac{k_{-2}\theta_{V}}{k_{-1}\theta_{V}+k_{2}(1-\theta_{V})}$$

or

(A23) $$\frac{\theta_{V}{}^{2}}{{\left(1-\theta_{V}\right)}^{2}}-\frac{2k_{1}{}_{N}C_{N2O}}{k_{-2}}\frac{\theta_{V}}{\left(1-\theta_{V}\right)}-\frac{2k_{1}{}_{N}C_{N2O}k_{2}}{k_{-1}k_{-2}}=0$$

Designating $B=k_{1}{}_{N}C_{N2O}/k_{-2}$ one can obtain the following quadratic equation:

(A24) $$\frac{\theta_{V}{}^{2}}{{\left(1-\theta_{V}\right)}^{2}}-2B\frac{\theta_{V}}{\left(1-\theta_{V}\right)}-2B\frac{k_{2}}{k_{-1}}=0$$

Its solving results in:

(A25) $$\frac{\theta_{V}}{\left(1-\theta_{V}\right)}=B+\sqrt{B+2Bk_{2}/k_{-1}}$$

One can express *θ_V_* and (1 − *θ_V_*) from Equation (A25):

(A26) $$\theta_{V}=\frac{B+\sqrt{B+2Bk_{2}/k_{-1}}}{1+(B+\sqrt{B+2Bk_{2}/k_{-1}})}$$

(A27) $$(1-\theta_{V})=\frac{1}{1+B+\sqrt{B+2Bk_{2}/k_{-1}}}$$

Substitution of Equation (A27) to the equation of the rate for stage 1 results in:

(A28) $$\begin{array}{l} R_{N2O}=2r_{1N}=2k_{1}{}_{N}C_{N2O}(1-\theta_{V})=\frac{2k_{1}{}_{N}C_{N2O}}{1+B+\sqrt{B+2Bk_{2}/k_{-1}}}=\\ \frac{2k_{1}{}_{N}C_{N2O}}{1+k_{1}{}_{N}C_{N2O}/k_{-2}+\sqrt{k_{1}{}_{N}C_{N2O}/k_{-2}+2k_{1}{}_{N}C_{N2O}k_{2}/k_{-1}k_{-2}}}=\\ \frac{2k_{1}{}_{N}C_{N2O}}{1+\sqrt{2k_{1}{}_{N}C_{N2O}k_{2}/k_{-1}k_{-2}}\sqrt{1+k_{-1}/k_{2}}+k_{1}{}_{N}C_{N2O}/k_{-2}} \end{array}$$

In the case $k_{1}{}_{N}C_{N2O}<<k_{-2}$, i.e., stage of N_2_O adsorption is rate-determining, then Equation (A28) can be reduced to:

(A29) $$R_{N2O}\approx \frac{2k_{1}{}_{N}C_{N2O}}{1+\sqrt{2k_{1}{}_{N}C_{N2O}k_{2}/k_{-1}k_{-2}}}$$

## Appendix D. Determination of the Order of deN_2_O Reaction Rate on FeO_x_/CeO_2_

We used the standard procedure of linearization for logarithmic dependence of the reaction rate on the logarithm of concentration (Figure A1) to determine the order of reaction for N_2_O. Data of the rates were obtained at two concentrations of N_2_O (0.15% and 1.0%) and three temperatures (600 °C, 650 °C, and 700 °C). The calculated order of the reaction (*n*) in this temperature interval was close to 1: *n* = 0.94 (700 °C), *n* = 1.00 (650 °C), *n* = 0.91 (600 °C). We did not reveal any dependence of *n* on the temperature and consider that observed dispersion is due to an error in reaction rate value measuring. The average value of *n* in this temperature interval is 0.97.
